# Independence from industry cannot be compromised

**DOI:** 10.1080/21614083.2017.1393296

**Published:** 2017-09-19

**Authors:** Graham T. McMahon

**Affiliations:** ^a^ Office of the CEO, Accreditation Council for Continuing Medical Education (ACCME), Chicago, IL, USA

Collaboration between clinicians and the pharmaceutical and medical device industry has led to many of the medical advances in the past century. When managed appropriately, relationships between clinicians and pharmaceutical companies benefit patients and enhance the practice of medicine. However, the authors’ recommendations for facilitating industry engagement in clinician education in Europe, if implemented, would cause serious, negative consequences for the quality and integrity of clinician education, professionalism, and self-regulation, and would erode the public’s trust in our profession.

Though industry and medicine share the overall goal of improving health, their interests and obligations can and do diverge. Medicine has a primary responsibility to put the needs of patients first. Accredited continuing medical education (CME) holds that same responsibility. In contrast, corporate entities have a responsibility to their shareholders and other vested stakeholders to thrive as businesses and maximise returns on investment. Thus, pharmaceutical employees are fiduciary to their company’s business interests, and are often incentivised to increase their company’s profits. There is a large volume of evidence that incentives provided to clinicians in educational environments (such as gifts and compensation for speaking engagements or for programme attendance) can and do lead to corrupt practices even though many clinicians consider themselves immune to these marketing tactics [1]. These inappropriate incentives have resulted in the misuse of educational environments to promote products and to influence prescribing behaviour in ways that adversely affect patients, public health, and healthcare costs.

Concerns about physicians’ relationships with industry have continued to grow as evidence has accumulated about the influence of such relationships on physician practice [2]. A consensus has emerged in the USA that recognises the enormous value of maintaining strong relationships between medicine and industry, notably in research and innovation, but equally recognises the need for circumspection where industry influence on physician education is concerned. Evidence demonstrating the influence of commercial manufacturers on clinician behaviour led the Accreditation Council for Continuing Medical Education (ACCME), the American Medical Association, and others to adopt guidelines related to industry funding of these programmes and the participation of speakers who have industry relationships [3,4].

## Separating promotion from education

The Standards for Commercial Support: Standards to Ensure Independence in CME Activities℠, created in 1992 and updated in 2004, are designed to ensure that accredited CME serves the needs of patients and not the interests of pharmaceutical or device manufacturers.

While drug/device companies are allowed to provide funding for accredited CME, this funding occurs within strict parameters. Companies are prohibited from having any influence over faculty or content; cannot pay attendees or faculty for travel, registration, or honoraria; and cannot influence who can attend. Industry representatives can participate in accredited education in very limited scenarios designed to ensure independence while facilitating the free flow of scientific discourse. This framework accommodates some of the concerns expressed by the authors – but without compromising independence, and data suggest that this framework is working to safeguard accredited CME from commercial bias [5].

The Standards reflect the consensus that has emerged about the need to maintain tight restrictions on industry involvement in education. The ACCME Standards are congruent with US government, institutional, and industry guidelines, and have become a common, interprofessional standard, adopted across the professions of medicine, nursing, pharmacy, optometry, and others in this country. These standards have also gained international recognition; they have been adopted by the European Board for Accreditation in Cardiology (EBAC), and by accreditors in Canada, Oman, and Qatar. The European Accreditation Council for CME (EACCME) standards approximate to those of the ACCME in several respects.

Industry guidelines, including the Pharmaceutical Research and Manufacturers of America’s (PhRMA) guidelines that were published in 2002 and updated in 2009, largely conform to these Standards. Notably, the 2009 PhRMA guidelines restrict companies from developing the direct relationships with prescribers that the authors advocate for Europe [6].

As a result of the widespread endorsement of the Standards, the US government has recognised the value of CME and engaged the CME community in public/private partnerships to improve public health. For example, the US Food and Drug Administration (FDA) leveraged the accredited CME community to deliver prescriber education about opioids. [7] These opportunities would not have happened without the CME community’s demonstrated ability to maintain independence from industry.

None of this is meant to imply that pharmaceutical companies could not and cannot ever be effective educators or that all of their outreach efforts are counter to the public interest: these companies are not the enemy of our profession, and we rely on them and their integrity to discover and produce the medicines and devices that promote health and wellbeing, and to participate in disease awareness and quality improvement initiatives. Nevertheless, the potential that these companies might compromise the independence of information being shared with prescribers and clinicians is cause enough to exclude them from direct involvement in accredited CME. In order for the professional education community to maintain its credibility and its accountability to the profession and the public, it is critical to maintain a clear, unbridgeable separation between accredited education and industry education, promotion, and marketing.

## An international trajectory toward transparency and accountability

As clinician education becomes increasingly global, the need for harmonisation of education standards for independence has become a cross-national priority. If European educational providers adopted the practices suggested by the authors, they would be effectively excluded from participating in international equivalency and credit-exchange standards. The balance of risk and harm favours a conservative approach that should highly restrict the participation of industry in clinician education.

Clinicians are expected to provide safe, effective, cost-effective, compassionate care based on best practice and evidence, not on promotion. This professionalism is the basis of medicine’s contract with society. It demands placing patients’ interests first, setting and maintaining standards of competence and integrity, and providing expert health advice to society. Essential to this contract is the trust of the public and clinicians, which depends on the integrity of each clinician and the profession as a whole. To fulfil our obligations, we must continue to ensure that accredited CME offers clinicians a protected space to learn and teach without commercial influence.
